# Advances in Bract Coloration: Diversity, Pigment Synthesis, and Regulatory Mechanisms in Ornamental Plants

**DOI:** 10.3390/plants14142155

**Published:** 2025-07-13

**Authors:** Xiaoyang Li, Yang Liu, Zhiquan Cai, Yiwei Zhou

**Affiliations:** 1 School of Agricultural and Biological Engineering, Foshan University, Foshan 528000, China; 2 Guangdong Provincial Key Laboratory of Ornamental Plant Germplasm Innovation and Utilization, Environmental Horticulture Research Institute, Guangdong Academy of Agricultural Sciences, Guangzhou 510640, China; 3 College of Architectural Engineering, Shenzhen Polytechnic University, Shenzhen 518055, China

**Keywords:** bract coloration, ornamental plants, anthocyanins, betalains, regulation, pigment biosynthesis

## Abstract

Bract coloration in ornamental plants is a complex trait governed by diverse pigments (chlorophylls, anthocyanins, betalains, and carotenoids), their biosynthetic pathways, and regulatory networks. While previous research has primarily focused on floral pigmentation, studies on bract coloration—particularly in species where bracts serve as the primary ornamental feature—have received less attention until recent advances. This review synthesizes current understanding of bract color diversity, pigment biochemistry, and molecular regulation in key species including *Bougainvillea*, *Euphorbia pulcherrima*, *Anthurium andraeanum*, *Curcuma alismatifolia*, and *Zantedeschia hybrida*. Anthocyanins predominantly contribute to red-to-purple hues, while betalains generate red, purple, or yellow coloration through differential accumulation of betacyanins and betaxanthins. Developmental color transitions are mediated by chlorophyll degradation and carotenoid dynamics. The spatiotemporal regulation of pigment accumulation involves coordinated interactions between key structural genes (*CHS*, *DFR*, *ANS* for anthocyanins; *DODA*, *CYP76AD1* for betalains), transcription factors (MYB, bHLH, WRKY), and plant growth regulators (BAP, GA, MeJA). Despite these advances, significant knowledge gaps remain in genetic inheritance patterns, epigenetic regulation, cross-pigment pathway crosstalk, and environmental modulation. Future research directions should integrate multi-omics approaches, wild germplasm resources, and gene-editing technologies to develop novel breeding strategies for bract color improvement.

## 1. Introduction

Bracts, as accessory organs of flowers or inflorescences, exhibit remarkable morphological diversity in color, size, and shape, while fulfilling diverse ecological functions [1]. Bracts are modified leaves that are considered to possess certain photosynthetic capabilities [1]. For instance, the albino bracts of *Davidia involucrata* exhibit limited photosynthetic activity, with their net photosynthetic rate, stomatal conductance, and transpiration rate being 96.38%, 84.49%, and 82.95% lower, respectively, compared to leaves [2]. However, exceptions exist under specific conditions; in cotton, bracts demonstrate superior photosynthetic performance, higher chlorophyll and rubisco content, and greater net photosynthesis than leaves under water stress [3]. In contrast, brightly colored ornamental bracts lack chloroplasts and, thus, are non-photosynthetic [4,5].

Beyond protecting flowers, fruits, or seeds from herbivory, bracts may deter predators through warning signals or camouflage [6]. This morphological and functional diversity results from natural selection, enabling adaptation to various ecological niches and fostering complex interactions within ecosystems [1,7]. For instance, in *Castilleja coccinea*, bract color polymorphism is linked to reproductive trade-offs: scarlet individuals exhibit higher seed production, whereas yellow individuals benefit from reproductive assurance, collectively maintaining population polymorphism [8]. Similarly, in species such as Davidia involucrata, bract color variation correlates with reproductive functions (e.g., pollen protection), enhancing pollen viability by up to 662% [9]. The large bracts of hummingbird-pollinated plants have been shown to attract hummingbirds [10]. Additionally, in *Thunia alba*, large bracts serve a protective role by deterring nectar robbers and enhancing the plant’s reproductive fitness [11].

Brightly colored and large bracts also play a crucial role in pollinator attraction [12]. However, their ecological significance extends beyond pollination. Bracts provide essential protection for reproductive structures, such as shielding flowers from harmful ultraviolet (UV) radiation, which can impair reproductive organs and reduce seed yield [13]. This protective function is particularly critical in habitats with high environmental stress [1]. In addition to their resistance to environmental stressors, bracts can also protect seeds and nectar from predation [14].

**Figure 1:**
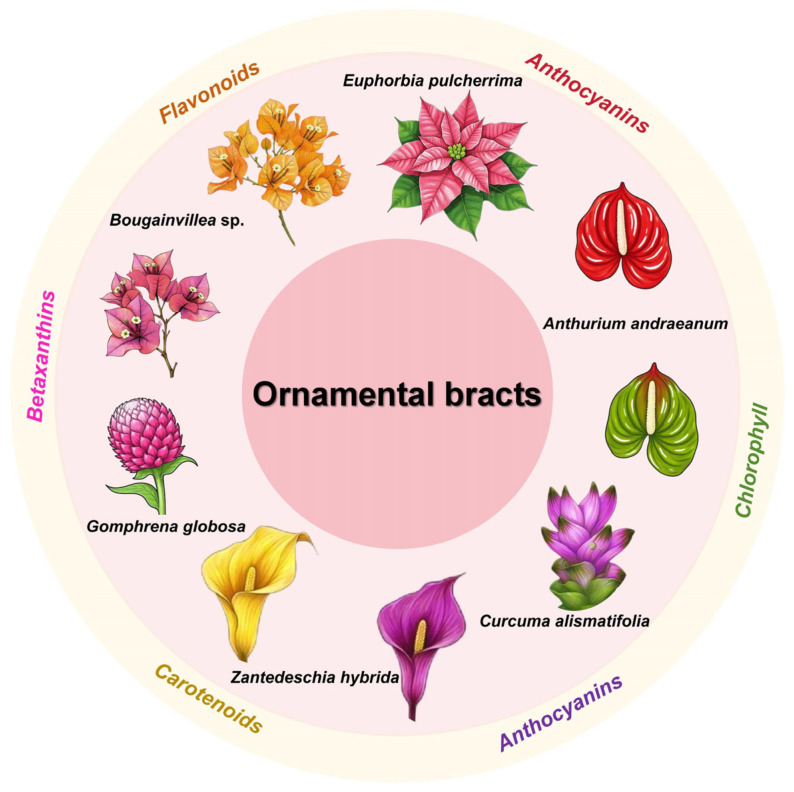
Major pigment composition in ornamental bracts of key ornamental plant species.

Additionally, bracts serve as the primary ornamental feature in many horticultural species. Although flower coloration has been extensively studied [15,16], research on ornamental bract pigmentation remains limited. Recent advances have been made in understanding bract color traits in key ornamental species, including *Bougainvillea*, *Euphorbia pulcherrima*, *Anthurium andraeanum*, *Curcuma alismatifolia*, and *Zantedeschia hybrida*, among others. Figure 1 shows the main pigments in the ornamental bracts of these key plant species.

## 2. Diversity of Color Phenotypes and Key Pigments in Ornamental Plants Bracts

### 2.1. The Color Phenotypic Diversity of Ornamental Plant Bracts

Bract coloration exhibits remarkable diversity across species and cultivars, with significant variation in both phenotypic expression and underlying pigments. In *Bougainvillea*, bracts display a wide color spectrum including red, purple, magenta, white, green, and orange [17,18,19,20]. Similar diversity is observed in *Curcuma alismatifolia* (white, pink, red, purple) [21], *Euphorbia pulcherrima* (red, pink, white, orange) [22,23,24], *Anthurium andraeanum* (red, pink, purple, white, green) [15,16,17,18,19,20,21,22,23,24,25,26,27,28,29], and *Zantedeschia hybrida* (black, purple-red, red, orange, yellow, pink, white, gold) [30,31]. Other species with colorful bracts include *Gomphrena globosa* (orange, red, purple) [32], *Globba* (white, pink, red, purple) [33,34], *Musella lasiocarpa* (yellow and red) [35], *Alpinia hainanensis* (pink) [36], *Telopea speciosissima* (white, green, red) [37], *Heliconia* (yellow, orange-yellow, orange, pink, red) [38,39], *Guzmania* (red) [40], and *Calathea crotalifera* (yellow and red) [41].

### 2.2. The Influence of Pigment Distribution and Cellular Structure on Bract Coloration

In most cases, petal pigments are primarily localized in the upper epidermal cells, though they may also occur in the palisade and lower epidermal tissues of darker-colored petals [42,43]. Notably, green petals are exceptionally rare in flowering plants [44]. In contrast, bracts of many plant species exhibit green pigmentation, while those displaying color diversity are often cultivated as ornamental specimens. The coloration of most ornamental bracts predominantly results from pigments concentrated in epidermal and parenchyma cells. For instance, anthocyanins are distributed in both upper and lower epidermis as well as adjacent mesophyll cells (including palisade and spongy tissues) [45]. Similarly, studies on *Zantedeschia* cultivars demonstrate that pigments responsible for bract coloration are mainly localized in epidermal and parenchyma cells [30].

Interestingly, beyond pigment composition itself, epidermal cell morphology significantly influences color presentation. Compared to flat cells, conical epidermal cells enhance light penetration and pigment absorption, thereby intensifying floral coloration [46]. Ornamental bracts exhibit interspecific variation in epidermal cell morphology. Microscopic analysis of 27 colored *Zantedeschia* spathes revealed uniformly flattened epidermal cells in all cultivars [30]. Conversely, in *Davidia involucrata*, pink bracts display irregularly shaped upper epidermal cells with higher anthocyanin content, whereas white bracts feature flatter cells with lower pigment concentrations [47]. Noda et al. documented that when magenta *Antirrhinum* mutants turned pink, their conical epidermal cells flattened—a transformation regulated by MYB-family transcription factors [48]. Despite these findings, research on the cellular mechanisms underlying coloration in ornamental bracts remains limited and warrants further investigation.

### 2.3. Key Pigments and Their Mechanisms in Bract Coloration

The primary pigments responsible for this coloration include chlorophylls, betalains, flavonoids (particularly anthocyanins), and carotenoids, with their relative contributions varying significantly among species and cultivars. Betalains play a dominant role in *Bougainvillea* and *Gomphrena globosa*, where betaxanthins and betacyanins produce yellow and red hues, respectively, with red bracts containing significantly higher betacyanin concentrations than yellow ones [18,49,50]. Anthocyanins are the primary pigments in *Curcuma alismatifolia* [21,51], *Globba* [33,34], *Anthurium andraeanum* [26], *Alpinia hainanensis* [36], and *Zantedeschia hybrida* [30]. Specific anthocyanins identified include cyanidin-3-*O*-glucosylrutinoside and cyanidin-3-*O*-rutinoside-5-*O*-glucoside in *Alpinia hainanensis* red bracts [36], malvidin 3-rutinoside, delphinidin-3-*O*-rutinoside, and peonidin-3-*O*-rutinoside in *Curcuma alismatifolia* [21], and cyanidin derivatives in *Zantedeschia hybrida* purple-red bracts [30]. Furthermore, anthocyanin accumulation patterns in *Anthurium andraeanum* ‘Sonate’ mutants exhibit distinct phenotypic correlations: the dark-green mutant displays elevated anthocyanin levels in bracts, while the reddish-brown mutant shows maximal anthocyanin accumulation in leaves. In contrast, chlorotic and albino mutants demonstrate significantly reduced anthocyanin content [28]. In *Euphorbia pulcherrima* bracts, cyanidin and pelargonidin derivatives determine deep-red and orange-red coloration, respectively [22,24].

Pigment accumulation and color manifestation in bracts are significantly influenced by developmental stages across multiple species. Young bracts typically exhibit high chlorophyll content, appearing green due to active photosynthesis. The dynamic balance between chlorophyll, flavonol, and carotenoid accumulation or degradation regulates bract color changes during development. In white *Bougainvillea* bracts, chlorophyll content progressively decreases during development, resulting in white coloration [17]. Carotenoid levels similarly influence color transitions at the bract tips of *Curcuma alismatifolia* [51]. *Zantedeschia* bracts demonstrate a remarkable chlorophyll increase (10-fold for Chl a/b) accompanied by 20% carotenoid reduction and chloroplast proliferation during post-maturation regreening [52,53,54]. *Euphorbia pulcherrima* exhibits stage-specific pigment profiles: chlorophyll dominates early development, while cyanidin-3-galactoside, cyanidin-3-glucoside, cyanidin-3-rutinoside, pelargonidin-3-glucoside, and pelargonidin-3-rutinoside become predominant pigments in later stages [55]. In *Calathea crotalifera*, the concentrations of chlorophylls, carotenoids, and anthocyanins in the bracts undergo dynamic changes during inflorescence development, contributing to their ornamental appearance [41]. The white bracts of *Davidia involucrata* primarily result from significantly reduced chlorophyll and carotenoid levels compared to green leaves [2]. However, some cultivars like *Zantedeschia pentlandii* ‘Best Gold’ maintain orange bracts through lutein, violaxanthin, and β-carotene accumulation [52].

Current research demonstrates that betalains and anthocyanins serve as the dominant pigments responsible for color diversity in ornamental bracts of major decorative species, while chlorophylls and carotenoids primarily function as auxiliary pigments that modulate coloration through stage-specific accumulation or degradation during bract development. As systematically summarized in Table 1, these pigment combinations create distinct color categories in bracts that serve as primary ornamental features across key horticultural plants.

### 2.4. Instrumental Methods for Pigment Detection and Quantification

In most studies investigating pigments in ornamental bracts, spectrophotometry and high-performance liquid chromatography-mass spectrometry (HPLC-MS) remain the primary analytical methods. Spectrophotometry serves as a fundamental method for extracting and quantifying various plant pigments, including chlorophylls, carotenoids, anthocyanins, and betalains, and has been widely employed in studies of ornamental bract pigmentation [17,25,26,27,28,38,41,47,50,51,53]. However, this approach typically only measures total pigment content without distinguishing specific structural variants. Recent advances in chromatography and mass spectrometry, coupled with reduced costs, have established high-performance liquid chromatography (HPLC) and its tandem mass spectrometry (MS) configurations as powerful tools for plant pigment analysis. For instance, researchers have identified diverse betalains (betaxanthins, betacyanins, and Σ-betalains) in *Bougainvillea* bracts using HPLC [55], Ion-Pair High-Speed Countercurrent Chromatography/Electrospray Ionization-Tandem Mass Spectrometry (IP-HSCCC/ESI-MS-MS) [56], high-performance liquid chromatography-Diode Array Detector-Tandem Mass Spectrometry (HPLC-DAD-MS/MS) [50], and High-Performance Liquid Chromatography-Diode Array Detector-Electrospray Ionization-Multistage Mass Spectrometry (HPLC-DAD-ESI-MS^n^) [32]. Similarly, anthocyanins and flavonoids have been characterized in *Euphorbia pulcherrima* bracts through HPLC and HPLC/MS [24,57,58], and in *Curcuma alismatifolia*, *Globba*, and *Alpinia hainanensis* bracts via Ultra-Performance Liquid Chromatography-Tandem Mass Spectrometry (UPLC-MS/MS) [21,34,36]. These chromatographic and mass spectrometric methods enable precise structural identification of pigments and determination of key chromogenic compounds. Notably, certain structurally unique pigments, such as acyl-oligosaccharide-linked betacyanins in *Bougainvillea* bracts, require specialized detection methods like IP-HSCCC/ESI-MS-MS [56]. However, studies on pigment separation and methodological development for ornamental bracts remain limited, highlighting the need for interdisciplinary collaboration.

The time-consuming processes involved in pigment extraction and analysis pose significant challenges for large-scale genetic investigations. To overcome this limitation, researchers have developed colorimeter-based predictive models. Studies on *Gerbera* flower coloration [59], *Caladium* leaf pigmentation [60], as well as bract color in *Curcuma alismatifolia* [21] and *Globba* [34] have revealed strong correlations between specific colorimetric parameters (a* values with anthocyanin levels; b* values with carotenoid concentrations), validating their utility as robust proxies for pigment content. Expanding these non-destructive, high-throughput approaches to additional ornamental bract species would significantly enhance the efficiency of pigment quantification in breeding and genetic studies.

## 3. Metabolic Pathway of Pigments in Ornamental Bracts

### 3.1. Chlorophyll Metabolism

Chlorophyll biosynthesis is a multi-step enzymatic process primarily occurring in plant chloroplasts. The key enzymes involved in chlorophyll synthesis include: glutamyl-tRNA reductase (HEMA1), which catalyzes the conversion of glutamate to 5-aminolevulinic acid (ALA); ALA dehydratase (ALAD), responsible for condensing ALA to form porphobilinogen (PBG); magnesium chelatase subunits (CHLH/CHLI), which insert Mg^2+^ to generate Mg-protoporphyrin IX; protochlorophyllide oxidoreductase (POR), facilitating the reduction in protochlorophyllide to chlorophyllide; and chlorophyll a oxygenase (CAO), a Rieske-type non-heme iron oxygenase that converts chlorophyll a to chlorophyll b [61,62]. The chlorophyll degradation pathway involves several critical enzymes: chlorophyllase (CLH), which hydrolyzes the phytol side chain of chlorophyll; pheophorbide a oxygenase (PAO), catalyzing the ring-opening of pheophorbide a to produce red chlorophyll catabolite (RCC); red chlorophyll catabolite reductase (RCCR), reducing RCC to colorless primary fluorescent chlorophyll catabolite (pFCC); and STAY-GREEN protein (SGR), regulating the degradation of chlorophyll-protein complexes [61,62]. 

In *Curcuma alismatifolia*, reduced expression of *CAO* in bracts leads to decreased chlorophyll content, resulting in red bracts, a finding consistent with the observed *CAO* expression pattern in white bracts [63]. Similarly, mutations in the *OsCAO1* gene in rice cause pale green leaves, demonstrating the crucial role of CAO activity in pigment accumulation [64]. Studies on *Bougainvillea* bracts identified multiple chlorophyll metabolism-related genes. Protochlorophyllide oxidoreductase A gene *BgPORA*, which catalyzes the reduction in protochlorophyllide to chlorophyllide, is highly expressed during early bract development but declines as bracts transition from green to white. Concurrently, increased expression of the chlorophyll degradation-related gene *BgSGR* (STAY-GREEN protein) accelerates chlorophyll breakdown, promoting bract whitening. Additionally, *BgPPH* (pheophytinase), *BgPAO* (pheophorbide a oxygenase, chloroplastic), and *BgRCCR* (accelerated cell death) contribute to chlorophyll degradation, further influencing bract color changes [17].

Dysregulation of photosynthetic pigment metabolism genes can disrupt chloroplast structure and impair pigment accumulation. In *Davidia involucrata* bracts, significantly lower levels of chlorophylls a, b, and carotenoids compared to leaves were attributed to downregulation of chlorophyll biosynthesis-related proteins (PPR and AARS) and elevated PAO activity [2]. Similarly, studies on *Guzmania* bracts identified chlorophyll synthesis-related enzymes (GTS and UROS) and the key degradation enzyme PPH, with PPH playing a pivotal role in chlorophyll breakdown [40].

### 3.2. Anthocyanins Biosynthesis

Anthocyanins are water-soluble pigments belonging to the flavonoid family that play a crucial role in determining plant coloration, exhibiting red, purple, and blue hues depending on environmental pH [16,65]. Their biosynthesis occurs through the phenylpropanoid and flavonoid pathways. The phenylpropanoid pathway involves key enzymes including phenylalanine ammonia-lyase (PAL), cinnamate-4-hydroxylase (C4H), and 4-coumarate-CoA ligase (4CL), while the flavonoid pathway requires chalcone synthase (CHS), chalcone isomerase (CHI), flavanone 3-hydroxylase (F3H), flavonoid 3′-hydroxylase (F3′H), flavonoid 3′5′-hydroxylase (F3′5′H), dihydroflavonol 4-reductase (DFR), and leucoanthocyanidin dioxygenase (LDOX/ANS) [15,16,66]. Subsequent modifications by glycosyltransferases (UFGT), acyltransferases (AT), and methyltransferases (OMT) through glycosylation, acylation, and methylation are essential for producing stable anthocyanin derivatives [15,67].

In *Curcuma alismatifolia*, significantly reduced expression of *DFR* (gene25158) and *ANS* (gene437) in white-bracted ‘Country Snow’ cultivars contrasts with their high expression in pink-bracted ‘Chiang Mai Pink’ varieties, correlating with anthocyanin accumulation patterns [63]. Similarly, low *ANS* and *DFR* expression in *Bougainvillea* redirects pigment synthesis towards carotenoids and betalains [18]. White bracts in *Euphorbia pulcherrima* result from downregulation of anthocyanin modification (*UGT79B10*) and transport (*GSTF11*) genes [23,55], while pink bracts in *Alpinia hainanensis* accumulate cyanidin and peonidin derivatives through upregulated *AhF3′5′H* and *AhUGT77B2*-mediated glycosylation and methylation [36]. Musaceae bract color diversity correlates with *F3′5′H* gene expansion, potentially regulated by transposable elements, methylation patterns, and expression levels [35]. CRISPR/Cas9-mediated *F3′H* knockout in *E. pulcherrima* reduces cyanidin and pelargonidin content, shifting bract color from red to orange-red without direct anthocyanin synthesis effects [24], whereas *DFR* overexpression enhances anthocyanin production in *Arabidopsis thaliana* [68].

Glycosylation (*UGT*) and transport (*GST*) genes critically influence anthocyanin stability, solubility, and cellular distribution. In *E. pulcherrima*, the hypervariable *GST* gene *Bract1*—phylogenetically similar to known anthocyanin transporters—contains a 4 bp repeat mutation causing white phenotypes. This mutation exhibits X-ray-induced instability, generating frequent color variants [69]. *Anthurium* spathe coloration depends on stage-specific expression of *CHI* and *CYP* genes, with *F3′H* expression strongly correlating with anthocyanin content and color intensity [70]. Conversely, low *CHI2* and *DFR1*/*DFR2* expression in *Zantedeschia* limits anthocyanin synthesis, producing pink and white spathes [31]. These findings demonstrate that tightly regulated expression of flavonoid biosynthesis genes governs anthocyanin accumulation and subsequent bract coloration.

### 3.3. Betalains Biosynthesis

Betalains are water-soluble pigments primarily found in Caryophyllales plants, including crops such as *Beta vulgaris*, *Chenopodium quinoa*, and *Amaranthus hypochondriacus*. These pigments are classified into red-violet betacyanins and yellow-orange betaxanthins [71]. The betalain biosynthesis pathway originates from tyrosine metabolism, involving several key enzymatic steps: (1) Tyrosine hydroxylase (TYRH) catalyzes the hydroxylation of tyrosine to form L-DOPA. (2) DOPA 4,5-dioxygenase (DOD) then cleaves L-DOPA to produce betalamic acid and cyclo-DOPA. (3) UDP-glucosyltransferase (UGT) mediates the glycosylation of cyclo-DOPA to form cyclo-DOPA-5-*O*-glucoside. (4) Finally, betalamic acid condenses with cyclo-DOPA-5-*O*-glucoside to yield betanin [72].

The *DODA* gene encodes a tyrosine decarboxylase that serves as a key regulatory enzyme in betalain biosynthesis by catalyzing the conversion of tyrosine to dopamine, representing the initial step in this pathway. Upregulation of *DODA* expression positively correlates with betalain accumulation in *Bougainvillea* bracts, particularly influencing betanidin and betacyanin production [50]. Conversely, reduced expression of *BpCYP76AD1* leads to decreased betalain synthesis, resulting in lighter bract coloration in *Bougainvillea* ‘Thimma’. Notably, heterologous expression of *BpCYP76AD1* and *BpDODA1* in tobacco significantly enhances anthocyanin accumulation in leaves [73].

The final step in betalain biosynthesis involves glucosyltransferases encoded by Betanidin *5GT*/*6GT* genes, which catalyze the transfer of glucosyl groups to specific hydroxyl positions (C5 or C6) of betanidin to form stable pigments such as betanin and isobetanin [19,67]. In white-bracted *Bougainvillea* ‘BXGZ’, reduced expression of Betanidin 5GT/6GT contributes to diminished betalain accumulation [18]. The color expression in *Gomphrena globosa* and *Bougainvillea* inflorescences is modulated by the ratio of betacyanins to betaxanthins (including arginine-, lysine-, and putrescine-betaxanthins), which is influenced by glycosylation patterns and acylation degrees [32]. Comparative genomic analyses reveal that betalain biosynthesis genes in *Bougainvillea* × *buttiana* ‘Mrs Butt’ primarily expanded through whole-genome triplication (WGT) events, while other cultivars underwent alternative expansion mechanisms such as tandem duplication. These duplication events increase gene copy numbers and expression levels, indirectly affecting bract coloration. Correlation analyses suggest that both betalains and flavonoids play major roles in color development in *Bougainvillea* × *buttiana* ‘Mrs Butt’ bracts [18]. Among 13 *BbCYPs* identified *BbCYPs*, *BbCYP40,* and *BbCYP220* show the strongest correlations with betalain and flavonoid accumulation, respectively [74]. Functional studies demonstrate that overexpression of *BpCYP76AD15* can induce betalain production in *Bougainvillea* callus cultures [75], further confirming the crucial role of cytochrome P450 enzymes in betalain biosynthesis.

### 3.4. Carotenoids Biosynthesis

Carotenoid biosynthesis initiates in plastids through the methylerythritol phosphate (MEP) pathway, which produces the precursors isopentenyl diphosphate (IPP) and dimethylallyl diphosphate (DMAPP). These substrates are subsequently converted into various carotenoids and xanthophylls via sequential enzymatic reactions, including condensation, isomerization/dehydrogenation, hydroxylation, and oxidation [15,76]. Although carotenoids play a key role in color modulation in the bracts of some ornamental plants, research on how carotenoid biosynthesis pathways regulate bract pigmentation remains limited. Current studies have identified several correlations between gene expression and carotenoid accumulation. In *Curcuma alismatifolia*, high expression levels of the carotenoid biosynthesis gene *ZEP* correlate with elevated carotenoid content in variegated bract regions [51]. Transcriptomic analysis of *Bougainvillea* bracts identified 35 carotenoid biosynthesis-related unigenes [75]. Moreover, another research found that *BbCYP85* showed a strong association with carotenoid content in *Bougainvillea* bracts [74]. In green spathes of *Anthurium*, the high expression of five carotenoid biosynthesis genes—*PDS* (Unigene54090), two *LUT5* (Unigene14810 and Unigene58790), *CrtR-b* (Unigene54067), and *ZEP* (Unigene8259)—coincides with substantial carotenoid accumulation [27]. However, these findings remain correlative, lacking functional validation through genetic manipulation or mechanistic studies.

## 4. Transcriptional Regulation

The formation of bract coloration is regulated by transcription factors (TFs) that bind to promoters of pigment biosynthesis genes. The MYB TF family, the largest among eukaryotes, serves as the most prevalent regulator of anthocyanin biosynthesis pathways [77,78,79,80]. In *Anthurium*, spathe color variation is determined by stage-specific expression of *CHI* and *CYP* genes, where AaMYB2 activate *CHS*, *F3H*, and *ANS* to promote anthocyanin accumulation [25,26,27,70]. The bHLH family, the second-largest plant TF group, forms functional complexes with MYB through conserved N-terminal domains to regulate downstream targets [81]. In *Zantedeschia*, bHLH1 potentially acts as a positive regulator of *ANS* expression to enhance anthocyanin synthesis [31]. Multi-omics analyses of *Curcuma alismatifolia* bracts identified MYB and bHLH TFs co-expressed with anthocyanin biosynthesis genes, suggesting their regulatory roles in pigment accumulation [21,63]. MYB and WRKY TFs also regulate betalain biosynthesis. In *Beta vulgaris*, BvMYB activates *BvDODA1* and *BvCYP76AD1* to promote betalain production [19]. The group IIb WRKY protein HmoWRKY40 transcriptionally represses *HmoCYP76AD1* to modulate betalain synthesis in pitaya [82], while HuMYB9 inhibits betalain biosynthesis by binding super-enhancers of *HuCYP76AD1*-1, *HuADH1*, and *HuDODA1* [83]. MADS-box (*BgAP1*, *BgFULL*) and SBP (*BgSPL16*, *BgCMB1*, *BgDEFA*) family genes are critical during *Bougainvillea* bract development from green to white [17].

Interestingly, several transcription factors involved in anthocyanin biosynthesis regulation in bracts have demonstrated the capacity to modulate petal pigmentation when ectopically expressed. For example, The anthocyanin biosynthesis pathway in white *Cymbidium* orchid petals was successfully reconstituted through biolistic co-transformation of *AaMYB1* (*A. andraeanum*) with its cognate *bHLH* partners [84]. Similarly, heterologous expression of *A. andraeanum AaMYB2* in tobacco leaves induced anthocyanin accumulation accompanied by upregulation of both early and late biosynthetic genes in the anthocyanin pathway [26]. Furthermore, expression of the bract-specific transcription factor *AaFUL1* from *Anthurium* in tobacco resulted in petal fading phenotypes [85]. These findings collectively suggest that the regulatory functions of MYB and FUL family transcription factors in anthocyanin biosynthesis exhibit considerable conservation across different plant tissues. However, the extent to which this functional conservation applies broadly to other transcription factor families remains to be systematically investigated through comprehensive molecular genetic studies.

## 5. Plant Growth Regulators

Phytohormones interact with pigment biosynthesis genes to modulate coloration [86]. MeJA, GA, and BAP significantly influence pigment accumulation in bracts. In Zantedeschia ‘Best Gold’, cytokinin (BAP) and gibberellin (GA_3_) delay bract regreening by suppressing chlorophyll metabolism, extending ornamental value [52,53,54]. Pitaya bracts treated with 1.0% chitosan/0.2% κ-carrageenan coatings plus 50 mg L^−1^ GA_3_ or 0.1 mM MeJA retain higher chlorophyll content and color stability [87]. In *Bougainvillea*, upregulated *JAZ* genes may mediate JA signaling to promote white bract formation, while their downregulation induces purple bracts [20]. These findings underscore the coordinated interplay between transcriptional regulatory networks and hormonal signaling pathways in determining bract pigmentation patterns.

## 6. Conclusions

Bract coloration in ornamental plants represents a complex phenotypic trait governed by intricate interactions among four major pigment classes (chlorophylls, anthocyanins, betalains, and carotenoids), their biosynthetic pathways, transcriptional regulation networks, and hormonal signaling cascades. The remarkable color spectrum observed in ornamental bracts stems from species-specific pigment profiles, where anthocyanins predominantly contribute to red-to-purple hues, while betalains produce red, purple, yellow, or orange coloration depending on the relative ratios of betacyanins to betaxanthins. Developmental color transitions are further modulated by chlorophyll degradation and carotenoid dynamics, as exemplified by the whitening process in *Bougainvillea* and regreening phenomenon in *Zantedeschia*. The metabolism of these pigments is precisely regulated through: (1) biosynthesis genes (e.g., *CHS*, *DFR*, and *ANS* for anthocyanins; *DODA* and *CYP76AD1* for betalains; *CAO* for chlorophylls), (2) degradation enzyme genes (e.g., *PAO* and *SGR* for chlorophyll catabolism), and (3) transcriptional control mediated by MYB, bHLH, and WRKY transcription factors in conjunction with plant growth regulators (BAP, GA, MeJA). These regulatory mechanisms collectively determine the spatiotemporal patterns of pigment accumulation.

Despite significant progress, including the functional validation of key genes influencing bract coloration (Table 2), critical knowledge gaps persist regarding (1) the complete genetic architecture underlying bract coloration, (2) epigenetic regulation mechanisms, and (3) cross-talk between different pigment pathways. Furthermore, current research remains largely limited to a few model species (e.g., *Bougainvillea*, *Zantedeschia*, *Euphorbia pulcherrima*, *Anthurium andraeanum*, and *Curcuma alismatifolia*) and their selected cultivars. This narrow focus presents substantial challenges in elucidating the universal principles governing pigment accumulation and genetic regulation across diverse ornamental species.

## 7. Prospects and Future Research Directions

Despite significant progress in understanding the molecular mechanisms underlying bract pigmentation, several key scientific questions remain unresolved. First, the genetic basis of pigment biosynthesis in bracts remains incompletely characterized, particularly with respect to systematic genetic mapping studies. Although four quantitative trait loci (QTLs) associated with bract color have been identified in globe artichoke [88], genetic linkage maps for most ornamental species have yet to be established, hindering the elucidation of inheritance patterns and the application of marker-assisted breeding.

The environmental regulation of pigment biosynthesis during bract development is also poorly understood. Factors such as temperature, light intensity, spectral quality, pH, sugar signaling, mineral elements, oxidative stress, and CO_2_ concentration can modulate the biosynthesis of these plant pigments [86,89]. For instance, in *Telopea*, chlorophyll, carotenoid, and anthocyanin levels in bracts are significantly influenced by light conditions, with shading treatments effectively maintaining pigment content [37]. Under blue light-deficient conditions, the bracts of *Guzmania* turn yellow [90]. Shading treatment significantly reduces chlorophyll, anthocyanin, and carotenoid contents in *Calathea crotalifera* bracts [41]. However, similar studies in other ornamental bracts remain scarce. Additionally, copigments such as flavonoids can enhance color intensity (hyperchromic effect) and induce spectral shifts (bathochromic shift) through interactions with anthocyanins [91]. A notable example is the formation of blue complexes in hydrangeas, where delphinidin-3-glucoside binds with 5-*O*-acylquinic acids in the presence of aluminum ions [92]. While flavonoids have been detected in bracts of *Bougainvillea* [50] and colored spathes of *Zantedeschia* [30], their specific roles in bract pigmentation require further investigation.

Multi-omics approaches remain underutilized in bract pigment research. Recent advancements, including chromosome-level genome assemblies for *Anthurium* [93], *Curcuma* [58,94], *Bougainvillea* [18], and *Zantedeschia elliottiana* [95], provide a robust foundation for systematically dissecting the molecular mechanisms of bract pigmentation. Future studies should leverage these genomic resources to employ high-throughput genetic analyses (e.g., GWAS, BSA-seq) for elucidating the genetic basis of bract color variation. Integrating transcriptomic, metabolomic, proteomic, and epigenomic data will further enable the construction of comprehensive gene regulatory networks, revealing coordinated control across pigment biosynthesis pathways.

Furthermore, current research predominantly focuses on cultivated varieties, with limited exploration of unique color variations in wild genetic resources. Expanding the collection and evaluation of wild germplasm, coupled with comparative genomic analyses, may uncover novel regulatory genes in pigment biosynthesis, thereby enriching the genetic toolkit for ornamental plant breeding. Functional validation of candidate genes using CRISPR/Cas9 and other gene-editing technologies will accelerate targeted breeding efforts for bract color improvement.

Based on current research advances, we propose the following priority areas for future investigation of ornamental bract coloration:

(1) Comparative pigment accumulation mechanisms: Systematic comparison of pigmentation processes between ornamental bracts, vegetative bracts, leaves, and petals to identify tissue-specific regulatory patterns.
(2) Environmental regulation of coloration: Molecular-level characterization of bract color variation in response to abiotic factors (light intensity, temperature, water availability) and biotic stresses.
(3) Copigmentation effects: Comprehensive analysis of copigment-pigment interactions and their impacts on color stability and diversity in ornamental bracts.
(4) Multi-omics integration: Combined application of genomics, transcriptomics, metabolomics, and proteomics with forward genetics approaches to elucidate key coloration factors and their regulatory networks.
(5) Advanced pigment separation technologies: Development of novel techniques for precise isolation and identification of key chromogenic compounds in bracts.
(6) High-throughput phenotyping systems: Develop efficient systems for detecting or predicting pigment content in ornamental bracts to enhance the phenotypic screening efficiency of bract pigment content for large-scale breeding programs.
(7) Molecular breeding tools: Development of functional markers through high-throughput sequencing and association mapping in diverse genetic populations to enable marker-assisted selection.
(8) Genetic transformation systems: Establishment of efficient, stable transformation protocols for targeted modification of coloration pathways in ornamental bract species.

## Figures and Tables

**Table 1:** Color categories of main ornamental bracts in different plants and their primary contributing pigments.

Species	Bract Color	Primary Pigment	References
*Davidia involucrata*	white	very low chlorophyll and carotenoid content	[2]
*Bougainvillea*	red, purple, magenta, white, purple, orange, green, yellow	betalains (betaxanthins, betacyanins), chlorophyll, carotenoids, anthocyanins, flavonoids	[17,18,19,20]
*Euphorbia pulcherrima*	red, pink, orange, coral, white, green, and brown	anthocyanins (cyanidin derivatives, pelargonidin derivatives)	[22,23,24]
*Anthurium andraeanum*	pink, red, puple	anthocyanins (cyanidin 3-rutinoside, pelargonidin 3-rutinoside)	[25,26,28]
*Anthurium andraeanum*	white	proanthocyanins or no anthocyanins	[27]
*Anthurium andraeanum*	green	chlorophyll, carotenoids	[27]
*Curcuma alismatifolia*	white, pink, red, purple	anthocyanins, chlorophyll	[21,51]
*Zantedeschia hybrida*	black, red, orange, yellow, pink, white, purplish red	anthocyanins (cyanidin and pelargonidin), flavonol	[30,31]
*Gomphrena globosa*	orange, red, purple	betaxanthins, betacyanins, Σ-betalains	[32]
*Globba* spp.	white, pink, red, purple	anthocyanins	[34]
*Alpinia hainanensis* ‘Shengzhen’	Pink	anthocyanins (cyanidin, pelargonidin, peonidin, petunidin)	[36]
*Telopea speciosissima*	white, green, red	chlorophyll, anthocyanins, carotenoids	[37]
*Heliconia*	Orange-yellow	Anthocyanins	[38]
*Guzmania*	red	flavonoids, anthocyanins (cyanidin chloride, pelargonium chloride)	[40]
*Calathea crotalifera*	red	Chlorophyll, carotenoids, anthocyanins	[41]
*Calathea crotalifera*	yellow	Chlorophyll, carotenoids	[41]
*Zantedeschia pentlandii* ‘Best Gold’	golden yellow	carotenoids: lutein, violaxanthin, β-carotene	[52,53]

**Table 2:** Recently cloned and functionally validated genes in ornamental bracts.

Species	Isolated Gene	Regulatory Mode	Methods	Effects on Pigment Accumulation	References
*Euphorbia pulcherrima*	*F3′H*	Positive	CRISPR/Cas9 gene editing	Reduced content of cyanidin and pelargonidin	[24]
*Euphorbia pulcherrima*	*DFR*	Positive	Heterologous transformation in Arabidopsis	Increased anthocyanin accumulation	[68]
*Bougainvillea*	*BpCYP76AD1* and *BpDODA1*	Positive	Co-transient transformation of two genes with MjcDOPA5GT in tobacco leaves	Enhanced betalain content in tobacco leaves	[73]
*Bougainvillea*	*BpCYP76AD15*	Positive	Homologous overexpression in callus	Betalain accumulation in callus tissues	[75]
*Anthurium andraeanum*	*AaMYB2*	Positive	Heterologous overexpression in tobacco	Increased anthocyanin levels in tobacco leaves	[26]
*Anthurium andraeanum*	*AaFUL1*	Negative	Heterologous overexpression in tobacco	Petal color fading	[85]
